# A Systematic Review of the European Rapid Alert System for Food and Feed: Tendencies in Illegal Food Supplements for Weight Loss

**DOI:** 10.3389/fphar.2020.611361

**Published:** 2021-01-26

**Authors:** Dorottya Koncz, Barbara Tóth, Orsolya Roza, Dezső Csupor

**Affiliations:** ^1^ Department of Pharmacognosy, University of Szeged, Szeged, Hungary; ^2^ Medical School, Institute for Translational Medicine, University of Pécs, Pécs, Hungary

**Keywords:** food supplements, rapid alert system for food and feed, illegal medicines, obesity, weight loss

## Abstract

**Background**: Slimming products represent a dynamically growing group of food supplements worldwide. The efficacy of safely usable natural ingredients is usually below consumers’ expectations. Certain manufacturers add unauthorized or prohibited ingredients to weight loss supplements in order to increase their efficacy. Hence, many of these products are adulterated and may pose a risk to the consumers’ health.

**Aims**: The aim of our work was to give an overview on natural ingredients used in slimming products, to summarize the frequently used synthetic adulterants and also to assess the trends of adulterated and illegal food supplements in the European Union based on the warnings of the Rapid Alert System for Food and Feed (RASFF) in the time period of 1988–2019.

**Methods**: Reports between 1988–2019 were extracted from the RASFF portal on January 1, 2020. Each entry was individually reviewed.

**Results**: 2,559 records of food supplements with quality problems were identified in the RASFF, several of which [319 (12,5%)] were marketed to facilitate weight loss. 202 (63,3%) contained unapproved, synthetic drug ingredients. The major adulterant (113 of 319, 35.4%) was DNP (2,4-dinitrophenol), whereas sibutramine was the second most frequent adulterant agent (69 products, 21,6%) between 1988 and 2019.

**Conclusion**: The number of approved medicines for the indication of weight loss is relatively low and their efficacy (and also that of the natural ingredients) is limited. Therefore, a significant number of weight loss supplements is adulterated to satisfy patients’ expectations. Hence, these products may cause serious adverse effects in sensitive patients.

## Introduction

Obesity is an emerging health problem worldwide, the number of affected people doubled from 1980 to 2008 (Finucane et al., 2011). Not only the incidence of obesity has been growing, but the world’s population’s mean BMI has also been increasing by a significant 0.4–0.5 kg/m^2^ in each decade (Finucane et al., 2011). Based on the World Health Organization’s (WHO) estimates, almost two billion adults had a body-mass index (BMI) ≥ 25.0 and of these adults more than 650 million people were classified as obese (BMI ≥30.0) in 2016 (World Health Organisation, 2018; Montan et al., 2019). Obesity does not only affect the United States, but it is becoming an epidemic also in Europe and even in several developing countries (Barness et al., 2007). According to a recently issued report, approximately 23% of the female and 20% of the male population of Europe were considered obese (World Health Organisation, 2019). If game-changing measures are not going to be applied, by the end of this decade, more than half of the world’s adult population will be obese or at least overweight (Kelly et al., 2008). Obesity is associated with many comorbidities, including cardiovascular (e.g. heart disease, hypertension) and cerebrovascular (e.g. stroke) ailments. The incidence of other severe chronic diseases, such as type 2 diabetes mellitus (T2DM) and degenerative musculoskeletal diseases (e.g. arthritis) are higher among overweight and obese people than among people with normal BMI. Moreover, Alzheimer’s disease and some malignant tumors affect more often obese people (World Health Organisation, 2018). Lifestyle interventions, involving dietary modifications and increased physical activity are of high importance in avoiding obesity; however, a high proportion of the individuals loses interest shortly after they have started their new lifestyle and return to their original one; therefore, the results are rarely permanent (Curioni and Lourenço, 2005). In the past century, many pharmacons have been approved to support weight loss; however, currently only three APIs and one combination product are available for this indication in the European Union (EU) (Tonstad et al., 2016). Several medicines are no longer available on the market because of safety concerns. Alternative methods are sought-after in the treatment of obesity. The most commonly used products for this purpose are food supplements, which are easily available on the market. However, these products are often counterfeited, containing illegal components. RASFF was set up to aid the national competent authorities in harmonizing their actions and informing one another in the control of food and feed that are posing serious risks (Rapid Alert System for Food and Feed (RASFF), 2020). RASFF is used in the European Union to help obtain the minimally required safety and quality of feed and food (i.e. safety and quality).

The goals of our study were to give an overview on the most widely used natural products, and on the safety profiles of available and withdrawn pharmacons that might be used to facilitate weight loss. We also aimed to assess their presence in food supplements on the European market associated with a warning released by RASFF and to summarize the trends from 1988 to 2019.

## Pharmacotherapy of Obesity

According to current guidelines, complex measures should be considered in obese patients and pharmacological therapy should only be used along other lifestyle modifications. Actions to address weight management should not only be applied in obese people (BMI ≥30), but also in patients with a BMI ≥27 kg/m^2^ if they already have metabolic syndrome or sleep apnea. The efficacy of pharmacotherapy should be assessed and reconsidered within 3 months of initiating the treatment (Yumuk et al., 2015), and it is highly recommended to complete pharmacotherapy with comprehensive lifestyle modifications including calorie deficit and pronounced physical activity.

A great deal of pharmacons previously used in weight management are no longer marketed because of safety issues. These drugs involve amphetamine derivatives, fenfluramine, lorcaserin, phenylpropanolamine, rimonabant, and sibutramine (Bray, 2014; Morris, 2018). The emerged safety concerns highlight that the potential side-effects (CNS-depressing effects, insomnia, toxicity, primary pulmonary hypertension) of pharmacons inducing weight loss should be monitored and assessed more thoroughly (Montan et al., 2019). Currently available medications for this purpose in the European Union are orlistat, liraglutide, naltrexone/bupropion and phentermine resinate (Yumuk et al., 2015; EMA 2020b; The State Institute for Drug Control (SUKL), 2020).

In the meantime, the popularity of food supplements for weight loss management and their market share have been increasing (Ríos-Hoyo and Gutiérrez-Salmeán, 2016). Unfortunately, some products are adulterated with synthetic substances, usually formerly approved weight loss compounds, which had already been withdrawn from the market. Hence, these food supplements can pose serious health risks for consumers.

### Medications of Obesity Available in the European Union

#### Orlistat

Orlistat (1) gained its marketing approval for obesity management by the European Commission (EC) in 1998 (Supplementary Table S1). It was the first selective, irreversible inhibitor of gastric and pancreatic lipase enzymes. Its mechanism of action involves the reduction of dietary fat lipolysis and absorption (Al-Suwailem et al., 2006). It is a prescription-only medicine in the European Union. Based on the results of a meta-analysis, orlistat decreased average body weight with 2.35 and 2.94 kg, at doses 60 and 120 mg, respectively (Li et al., 2005). Its use may be associated with clinically relevant mild-to-moderate gastrointestinal adverse effects (e.g. abdominal pain, diarrhea, fecal spotting, and steatorrhea). Apart from the above-mentioned minor side-effects, serious adverse reactions were also associated with the use of orlistat (e.g. subacute liver failure, cholelithiasis and cholestatic hepatitis). The safety of the chronic use of orlistat is highly questionable because it affects the absorption of other pharmacons and fat-soluble vitamins (Filippatos et al., 2008).

#### Liraglutide

Liraglutide acts as a glucagon like peptide-1 (GLP-1) receptor agonist and it was first used for the treatment of T2DM (Collins and Costello, 2019). Liraglutide was a promising pharmacon in the therapy of T2DM, because it improved the patients’ cardiovascular status and outcome (Marso et al., 2016). After being proved in human studies that GLP-1 analogues promote weight loss, it has become an approved drug for weight management. The mechanism of action of liraglutide involves appetite suppression and delayed gastric emptying (Mehta et al., 2017). Nevertheless, liraglutide was authorized by the European Medicine Agency (EMA) as an adjunct to a comprehensive lifestyle measures to induce weight loss (Supplementary Table S1; Christou et al., 2016; EMA, 2020b).

Liraglutide is available as an injection, but another GLP-1 receptor antagonist, semaglutide can be given *per os*. Semaglutide gained its EMA approval recently for the treatment of adults with insufficiently controlled T2DM to improve glycemic control (Gomez-Peralta and Abreu, 2019). Semaglutide is also monitored; therefore, rapid identification of safety concerns and unknown side effects are made possible (EMA, 2020a).

In human studies, the most common side effects associated with the intake of liraglutide included gastrointestinal symptoms (e.g. nausea and vomiting, risk of pancreatitis), and increased pulse rate (Marso et al., 2016). In a clinical trial, from randomization to the 20^th^ week, the mean weight loss in the intention-to-treat (ITT) population, was statistically significantly bigger in the liraglutide treated groups when compared to placebo. The effect was dose-dependent, daily doses of 1.2, 1.8, 2.8, and 3.0 mg liraglutide resulted in a weigh loss of 4.8, 5.5, 6.3, and 7.2  kg, respectively. Taking placebo resulted in a 2.8  kg weigh loss (Astrup et al., 2009).

#### Naltrexone-Bupropion

After subsequent clinical trials demonstrated its safety, a combination therapy, containing naltrexone (2) and bupropion (3) has been approved in the EU for weight management (Supplementary Table S1). Naltrexone acts as an opioid antagonist on the μ-opioid receptor and decreases appetite by inhibiting β-endorphin-mediated autoinhibition of POMC (pro-opiomelanocortin) neurons (Grossman et al., 2003; Cone, 2005; Greenway et al., 2009). The anorectic effects of the antidepressant bupropion is mediated via the stimulation of the activity of POMC cells in the arcuate nucleus of the hypothalamus (Caixàs et al., 2014). Significant weight loss was observed in participants assigned to the combination group after the end of a 56-week-long trial. Patients in the verum group received 32 mg of naltrexone and 360 mg bupropion daily. In groups showing the highest weight loss, the combination was applied for 28–36 weeks, mean change in bodyweight was −6.1 kg (Greenway et al., 2009). Based on the above mentioned evidence, this combination may serve as a possible treatment adjunct to lifestyle modifications by promoting satiety, reducing appetite, enhancing energy expenditure; therefore, it helps patients to achieve weight loss goals (European Medicines Agency, 2015; Sherman et al., 2016). Initially, the first clinical trials focused on the cardiovascular side effects of the combination (Sherman et al., 2016; Tek, 2016). The combination of naltrexone and bupropion is contraindicated in patients with uncontrolled hypertension; however, the possible risks of this combination on cardiometabolic parameters of the patients is not fully understood (Connolly et al., 1997; James et al., 2010). Based on the clinical trials, frequently occurring adverse effects in participants in the verum group were nausea, headache, constipation, dizziness, vomiting, and xerostomia (Vorsanger et al., 2016). Bupropion alone, and in combination with naltrexone increases the blood pressure; therefore, therapies in which bupropion is administered should only be initiated in patients whose blood pressure is well-controlled. Moreover, the patient’s blood pressure is to be checked regularly by either the patient itself or by a health care professional throughout the whole course of the treatment.

#### Phentermine

Phentermine (4) has great potential as a weight loss drug. It also acts on the POMC neurons of the hypothalamus by inhibiting the norepinephrine transporter (Narayanaswami and Dwoskin, 2017). Weigh loss of up to 6 kg have been reported by taking phentermine (15–30 mg/daily) (Lonneman et al., 2013). Phentermine had been used as an approved drug to overcome obesity in Europe since 1956 (Supplementary Table S1), and it was a frequently prescribed medicine for decades (Colman, 2005). However, in 2012 the EMA withdraw the marketing authorization of phentermine because of its side-effects. Based on the published evidence, the compound might affect the cardiovascular and the central nervous system when used for a long time. Moreover, the authority was concerned about the possibility of the use of the drug by obese adults for whom taking phentermine may have deteriorating effects (Shin and Gadde, 2013). Nevertheless, phentermine resinate is available in modified-release capsules in the Czech Republic based on the authorization of the national competent authority (The State Institute for Drug Control (SUKL), 2020). Phentermine was combined with topiramate (PHEN/TPM) to achieve weight loss goals and enhance quality of life of obese people. The combination was to be used once daily, but its approval was withdrawn in Europe in 2012, due to concerns regarding its safety (Shin and Gadde, 2013). Although, a 56-week-long trial conducted with a controlled release formulation of phentermine and topiramate (containing 15 mg phentermine and 92 mg topiramate) was proved to have an outstanding efficacy; however, it has to be noted that its use increased dose-dependently the occurrence of psychiatric and cognitive adverse events (Scheen and Van Gaal, 2014).

### Withdrawn Amphetamine Derivative-type Medications in the European Union

Amphetamine and its derivates (e.g. phenylpropanolamine, fenfluramine, dexfenfluramine) were used for obesity the first time in the 1930s (Bray and Greenway, 1999). These compounds are mainly derived from phenylethylamine from which neurotransmitters dopamine, epinephrine and norepinephrine are derived. The weight loss mechanism of action of amphetamine derivatives involves the stimulation of norepinephrine- and dopamine-release in the hypothalamic and limbic regions’ satiety centers (Bray, 1993). Amphetamine derivatives exert their anorexigenic effect for a few hours; but tolerance develops relatively fast within only a few weeks. Amphetamine use and abuse often result in cardiac complications (Bazmi et al., 2017). Amphetamine derivatives used as weight loss therapy were removed from the legal market in Europe due to safety concerns and the quick development of tolerance (Supplementary Table S2).

#### Phenylpropanolamine

Phenylpropanolamine (PPA, 5) is available without prescription, and commonly administered as an appetite suppressant for weight loss, and also in cases of cough and common cold (Kernan et al., 2000). PPA is chemically related to the amphetamine-like anorectic agents (Walker et al., 1996). Effects of PPA in weight loss is documented, but the exact mechanism of action is not fully elaborated (Wellman and Sellers, 1986).

Despite the fact that its safety and efficacy profile is controversial, the drug is still available in some European countries. There are safety concerns suggesting a link between the consumption of PPA and stroke (Kernan et al., 2000). In a clinical trial, complementary to a 5,023 kJ (1,200 kcal) diet, patients in the verum group took 75 mg of sustained release PPA for 6 weeks and the achieved weight loss was higher than in the non-treated placebo group (Schteingart, 1992). Patients in the verum group lost 2.59 kg, while the results in the placebo-receiving group was less pronounced (−1.07 kg). 36 patients of the original study were agreed to be enrolled in a further double-blind trial up to 20 weeks, and the difference remained significant (PPA −5.1; placebo −0.4 kg, *p* = 0.01). In spite of the more weight loss in the PPA group, patients did not report a greater anorexigenic effect. The authors of the article concluded that phenylpropanolamine can be used in combination with a diet based on calorie deficit to promote safe weight management.

#### Fenfluramine, Dexfenfluramine

(+)-Norfenfluramine (6), the active metabolite of prodrugs fenfluramine and dexfenfluramine, induces weight loss and it is a potent agonist on 5-hydroxytryptamine (5-HT_2C_) receptors (Porter et al., 1999; Fitzgerald et al., 2000). Both drugs, fenfluramine and its (S)-isomer, dexfenfluramine were used in monotherapy, the former one for short-term, and the latter one for long-term weight management, even if its long-term safety was not yet documented (Weintraub et al., 1992). The effects of dexfenfluramine were examined on obese women (n = 52) in a placebo-controlled, double-blind study. Patients in the dexfenfluramine group followed a 1,500 kcal/day diet and took 15 mg dexfenfluramine twice a day (Ditschuneit et al., 1996). After completing the 12-months-long trial, patients in the verum group lost 14.2 ± 2.20 kg, while patients in the placebo group lost only 4.92 ± 2.99 kg. The side-effects of dexfenfluramine are quite alarming though; based on a case-control study, it increased the prevalence of cardiovascular diseases, and its use was associated with pulmonary hypertension (Abenhaim et al., 1996). Therefore, because of safety concerns, both drugs, and the so called fen-phen formulation (combination of fenfluramine and phentermine) were withdrawn from the market in 1997 (Weintraub et al., 1992; Wadden et al., 1998; European Medicines Agency, 2003).

### Withdrawn Non-amphetamine Derivative Type Medications in the European Union

#### 2,4-Dinitrophenol

The compound 2,4-dinitrophenol (DNP, **7**) was initially applied in explosive mixtures, but in 1933 it was discovered that DNP causes significant weight loss, and soon it was marketed in slimming products (Tainter et al., 1933). DNP contributes to weigh management by increasing the basal metabolic rate (Cutting et al., 1933). However, serious adverse effects occurred so often that it was withdrawn from the market, and it was labelled as an ‘extremely dangerous’ drug (Supplementary Table S3; Tainter et al., 1934; McFee et al., 2004; Colman, 2007). The side-effects of DNP are associated with its mechanism of action: DNP induces a hyper-metabolic state of the body via uncoupling oxidative phosphorylation, and the excess energy becomes thermal energy in the mitochondria. Hyper-metabolite state is followed therefore with an uncontrolled thermogenesis causing hyperthermia and undesirable elevated body temperature associated with systemic responses (Tainter et al., 1935). After 1938, DNP was no longer prescribed and reports on severe side-effects did not occur, but it is assumed that the use of the compound has not been vanished completely, because case reports of DNP caused deaths still emerged after it has been withdrawn from the legal market (Colman, 2007). Today, DNP is sold illegally as a weight loss aid under a number of different names and its use is encouraged by reports of rapid and safe weight loss (McFee et al., 2004).

#### Rimonabant

Rimonabant (8), the first antagonist on the cannabinoid receptor type 1 (CB_1_-receptor) entered the European market in 2006 (Supplementary Table S3) (Rinaldi-Carmona et al., 1995). Initially, it was a promising medication, since several trials proved its effects on weight loss and it also improved several parameters of metabolic syndrome. A meta-analysis of RCTs evaluating the efficacy and safety of rimonabant (20 mg/day) found that the average weight loss in the treated group was 4.7 kg (4.1–5.3 kg), significantly higher, compared to the placebo group (Christensen et al., 2007). However, the use of rimonabant was linked to diverse psychiatric adverse events (e.g. anxiety, depression, and suicidal ideation); therefore, the EMA withdrew the market authorization of rimonabant in the EU in January 2009 (Sam et al., 2011).

#### Sibutramine

The antidepressant sibutramine (9) inhibits the reuptake of neurotransmitters serotonin (5HT)- and noradrenaline (NA). Apart from its original application later on it was found to reduce appetite (McNeely and Goa, 1998). In a 12-week-long study, the effects of sibutramine was similar to that of dexfenfluramine. Patients in both groups lost significant amount of weight (4.5 kg, and 3.2 kg, respectively). Sibutramine was used at daily doses ranging from 5 to 30 mg. Based on the results of this study, the optimum daily dose of the drug is 10–15 mg (Lean, 1997). After reports on increased diastolic and systolic blood pressure and pulse rate, concerns were raised regarding the safety of sibutramine (Sharma et al., 2009)*.* Hence, its safety was assessed in the so called Sibutramine Cardiovascular Outcomes Trial (SCOUT) (James, 2005). In this trial the harmlessness of sibutramine was evaluated on patients with a history of cardiovascular disease. As a result, the EMA concluded that the risk-benefit ratio was unfavorable for sibutramine and recommended to suspend all marketing authorizations for sibutramine-containing medicines in Europe (Williams, 2010). Sibutramine was approved in 2001, whereas its market authorization was suspended in 2010 (Supplementary Table S3; Ioannides-Demos et al., 2005).

#### Lorcaserin

Lorcaserin (10), an 5-HT_2C_ receptor agonist, was an approved drug for long-term treatment of obesity and it was intended to be used along with reduced-calorie diet and increased physical activity (Smith et al., 2010). In 2013 (Supplementary Table S3), only one year after its marketing approval, the marketing authorization holder officially notified the EMA’s Committee for Medicinal Products for Human Use (CHMP) about his wish to withdraw the marketing authorization for lorcaserin, because based on the CHMP’ opinion the benefits of lorcaserin—a medicine intended for helping to achieve weight control in obese and overweight patients—did not outweigh its risks (e.g. depression, valvulopathy) (European Medicines Agency, 2013). The weight loss achieved after a one year treatment with lorcaserin ranged from 4.5 to 5.8 kg in the published clinical trials when taking 10 mg lorcaserin once or twice daily (Fidler et al., 2011). Patients taking lorcaserin experienced a significant increased risk of depression (DiNicolantonio et al., 2014). Long-term use might be associated with increased cancer risk (LiverTox, 2012b).

## Food Supplements for Weight Loss

Considering the limited efficacy and unfavorable side-effect profiles of synthetic drugs, there is a high demand for alternative treatments like herbal products to induce weight loss (Bahmani et al., 2015). One further reason for turning to alternative preparations is the fear from the possible side-effects of synthetic drugs. However, natural origin does not guarantee safety, as it can be demonstrated on the example of ephedrine, a natural alkaloid of species of Ephedraceae having a remarkably high cardiovascular risk (Samenuk et al., 2002). It is a myth that the use of herbal substances are always safe and harmless, and it is important to highlight that herbal compounds can have an interaction with medicines and products of natural origin can cause adverse events as well (Pittler and Ernst, 2001; Dwyer et al., 2005; Poddar et al., 2011; Astell et al., 2013). Moreover, since the regulation and control of food supplements is less strict compared to medicines, the ratio of conterfeit or mislabbelled, potentially dangerous products might be higher.

According to a recent review, most popular natural ingredients marketed for weight management include chitosan, glucomannan, capsaicin, carnitine, and conjugated linoleic acid (CLA) (Wharton et al., 2020). In Europe, other popular herbal ingredients include *Camellia sinensis* (L.) Kuntze (Theaceae), *Garcinia cambogia* (Gaertn.) Desr. (Clusiaceae) and unroasted seed of *Coffea arabica* L. (Rubiaceae) (Barrea et al., 2019; Ríos-Hoyo and Gutiérrez-Salmeán, 2016; The Plant List, 2013). *Hoodia gordonii* (Masson) Sweet ex Decne. (Apocynaceae), *Stevia rebaudiana* (Bertoni) Bertoni (Compositae), *Acaciopsis rigidula* (Benth.) Britton and Rose (Leguminosae family, syn. of *Acacia rigidula*) also occurred frequently as constituent of weight loss products in the warnings of RASFF (The Plant List, 2013). In the following, we present an overview of the most popular ingredients of weight loss products, including some plants that were common constituents of products reported in the RASFF system.

### Chitosan

Chitosan, a polysaccharide composed of β-(1→4)-linked d-glucosamine and *N*-acetyl-d-glucosamine units, is formed by the deacetylation of chitin. This compound can be found in the animal kingdom (e.g. the exoskeleton of crustaceans and insects) (Mesa Ospina et al., 2015). It contributes to weight management by lowering the absorption of dietary fat and cholesterol, and it might also promote fat excretion leading to weight loss without dietary modifications (Pokhis et al., 2015). A meta-analysis of 14 RCTs studied the effects of chitosan on body weight, serum lipids and blood pressure (Moraru et al., 2018). The results indicated that by the use of chitosan as a food supplement for up to 52 weeks might promote weight loss (average −1.01 kg). Apart form its slight effects on the body weight, the consumption of chitosan was associated with improvements of serum lipid profile and a significant reduction blood pressure, both systolic and diastolic (−2.68 mmHg, and −2.14 mmHg, respectively).

Based on the published studies, the short-term use of chitosan is safe, but except for those with shellfish allergy (Waibel et al., 2011). Adverse effects include flatulence, constipation, indigestion, nausea, and heartburn (Gallaher et al., 2000). Chitosan might interact with warfarin and partially interferes with the absorption of the fat-soluble vitamins (i.e. vitamins A, D, E, and K); however, its effects on the fecal fat excretion are not fully proven (Huang et al., 2007; Jull et al., 2008).

### Glucomannan

The most commonly used type of glucomannan (GM) in weight loss products is extracted from tubers of *Amorphophallus konjac* K. Koch (Araceae) (Xiao et al., 2000, The Plant List, 2013). GM, a hemicellulose-type polysaccharide induces weight loss through several mechanism. It makes the absorption in the small intestine slower; however, reduces the transit time in the small intestine, because it increases the viscosity of the content; furthermore, GM increases energy loss via fecal excretion. GM induces satiety in several ways: the consumption of GM enhances mastication efforts, and after its consumption it delays gastric emptying; moreover, elevated levels of plasma cholecystokinin induces cephalic– and gastrointestinal-phase satiety signals (Gallaher et al., 2000; Ríos-Hoyo and Gutiérrez-Salmeán, 2016). A meta-analysis found that the daily consumption of GM (1.2–15.1 g/day) for 5 weeks improves the patient’ metabolic profile, but only slightly affects the body weight (WMD: −0.79 kg) (Sood et al., 2008). However, contrasting results were reported by Zalewski and Szajewska, 2015, claiming that in otherwise healthy overweight or obese adults, short-term use of GM may promote a slight weight loss, but it does not affect the BMI. The effects of GM on children are not enough to establish a firm conclusion. Mild gastrointestinal adverse effects (bloating, diarrhea) were associated with the use of GM (Keithley and Swanson, 2005)*.*

### Capsaicin

Capsaicin (11) and capsaicinoids are agonists of the TRPV1 (transient receptor potential vanilloid subfamily 1) receptor, and they mimic the effects of cold, which decreases the fat mass through the activation and recruitment of brown adipose tissue (Saito, 2015). Capsaicin affects the oxidation of lipid and influences energy expenditure (Whiting al., 2014; Ludy et al., 2012; Zheng et al., 2017). Food rich in capsaicin contributes to weight management by preventing obesity (Zheng et al., 2017). There are also reports that the body weight of healthy women who regularly use chili peppers is slightly reduced when compared to those who do not use chili (Henry and Emery, 1986; Yoshioka et al., 1998). A meta-analysis based on eight studies involving 191 participants concluded that patients who took 2 mg capsaicin before each meal consumed less calorie by an average of 74 kcal; therefore, capsaicin may help maintain weight by reducing total energy intake (Whiting et al., 2014). However, based on other literature data, the level of its effects on thermogenesis and fat oxidation is moderate and its long-term efficacy is questionable (Lejeune et al., 2003). A middle-aged man with normal BMI would lose approximately 0.5 kg over 6.5 years if he pursued a calorie deficit diet of 10 kcal and consumed hedonically acceptable doses of capsaicin (Ludy et al., 2012), whereas a weight loss of 2.6 kg over 8.5 years would be reachable if he was in a 50 kcal negative energy balance (Galgani and Ravussin, 2010; Hall, 2010). Capsaicin at acceptable doses is safe, although it might cause mild-to-moderate gastrointestinal side-effects, sweating, flushing, and rhinorrhea (Avesaat et al., 2016). In addition, capsaicin compounds can interfere with antihypertensive agents (Patanè et al., 2010).

### Carnitine

Carnitine (12) transports long-chain fatty acids (FAs) into the mitochondria for transformation, and energy is produced from these FAs via β-oxidation, and it also aids eliminate toxic compounds from the cell (NIH, 2017). L-carnitine is the isomer of carnitine that is used to enhance weight loss (Elmslie et al., 2006). Nine RCTs involving 911 patients were summed up and analyzed in a systematic review and meta-analysis (Pooyandjoo et al., 2016). Participants receiving carnitine (in doses varying from 1.8 g/day to 4 g/day) lost significantly more weight (−1.33 kg) and their BMI decreased significantly more (−0.47 kg/m^2^) than patients receiving the control treatment. The results revealed that the weight loss effects of carnitine diminished over time. l-carnitine is very well tolerated; at doses of up to 15 g daily and there were only a few, mild side effects like infrequent diarrhea, gastralgia and nausea (Goa and Brogden, 1987).

### Conjugated Linoleic Acid

Conjugated linoleic acid (CLA) and its isomers activate different nuclear receptors and, thus, they differentially regulate the expression of those genes that are related to lipid metabolism (Chin et al., 1994). The natural sources of are beef meat and dairy products, but it can also be found in dietary supplements (Schmid et al., 2006). A meta-analysis of human studies indicated that the effect of CLA on weight loss was superior to that of the placebo: median doses of 3.2 g was effective and reduced fat mass (Whigham et al., 2007). However, in certain studies, the association between CLA and weight loss was not observed (Wharton et al., 2020). Nevertheless, there is a clear need for further, larger human trials assessing the efficacy and safety of CLA dietary supplements (Whigham et al., 2004). In animal studies CLA interfered with glucose metabolism (e.g. increased insulin resistance in mice) and lead to a change in liver function inducing lipodystrophy; therefore, these effects should be evaluated in human studies as well to rule out any safety concerns (Clément et al., 2002).

### Green Coffee

Unroasted seed of *Coffea arabica* L. are good sources of chlorogenic acids (13) that are not present in roasted coffee because of their thermolability (Farah et al., 2008). The possible use of green coffee in weight management is related to its chlorogenic acid content (Shimoda et al., 2006). Chlorogenic acid-rich green coffee extracts reduce blood lipid and glucose levels, blood pressure, and reduce the risk of certain cardiovascular diseases (Sanlier et al., 2019). A meta-analysis of three RCTs involving a total of 142 participants revealed that by the consumption of green coffee extract (GCE) a significant reduction in body weight was achieved (Onakpoya et al., 2011b). The authors of the above-mentioned meta-analysis could not establish an effective dose for the extract. The grade of evidence of this analysis is moderate, and if more rigorous trials with longer duration are published, the efficacy and safety of GCE in weight management might become appraisable.

### Green Tea

The non-fermented leaves of *Camellia sinensis* (L.) Kuntze is green tea. Its main active compounds are catechin polyphenols, such as epicatechin, epigallocatechin, epicatechin-3-gallate and epigallocatechin-3-gallate (EGCG, 14). From these constituents, EGCG is the one in which green tea is the most abundant and this compound is the most important with respect to its pharmacological effects (Musial et al., 2020). Caffeine is also a major pharmacologically active ingredient of green tea (Jeukendrup and Randell, 2011). It was found that a combination of green tea and caffeine contributed to weight management in people who usually consume low amounts of caffeine. The effect was attributed to enhanced thermogenesis and fat oxidation (Dulloo et al., 1999). This might be explained by the caffeine content of tea, since high doses of caffeine elevates thermogenesis and fat oxidation and lowers leptin levels; therefore, it might have body weight reducing effects (Westerterp-plantenga et al., 2005). Moreover, catechins, especially EGCG, inhibits catechol-*O*-methyltransferase (COMT) and therefore enhances fat oxidation (Westerterp-plantenga et al., 2005). A meta-analysis of human studies carried out with green tea proved that EGCG-containing extracts have significant effect on weight loss and its maintenance (WMD: −1.31  kg; duration at least 12 weeks). When analyzing the data from studies in which high regular caffeine intake was recorded, the effect of caffeine intake on body weight was not significant. However, the studied population differed in these two groups, i.e. low caffeine intake was studied on Asian participants and moderate-to-high caffeine doses were studied in Caucasian people; therefore, the conflicting results might derive form the heterogeneity of the included studies (Hursel et al., 2009). Green tea extracts are common constituents of slimming products; however, there are concerns about the hepatotoxicity of extracts with high (>100 mg/day) EGCG content (Oketch-Rabah et al., 2020).

### *Garcinia cambogia*

The main acid compound of *Garcinia cambogia* (Gaertn.) Desr. is (-)-Hydroxycitric acid (HCA, 15). This compound have proven adenosine triphosphate (ATP) citrate lyase inhibitory effects (Watson et al., 1969). The inhibition of the above mentioned enzyme restricts the availability of acetyl coenzyme A (acetyl-CoA) units that are necessary for fatty acid synthesis and lipogenesis during a so called lipogenic diet when patients consume high amounts of carbohydrates (Kornacker and Lowenstein, 1965; Bressler and Brendel, 1966; Linn and Srere, 1979). The compound restrains the synthesis of FAs, lipogenesis, appetite, and therefore aids weight loss (Sullivan et al., 1972). Despite its promising mechanisms, clinical studies have shown controversial findings (Jena et al., 2002). Nine RCTs were analyzed in a meta-analysis which revealed a small but significant weight loss promoting effect of HCA when compared to placebo (Onakpoya et al., 2011a). The duration of the included studies varied from 2 to 12 weeks, and the participants took 1–2.8 g of HCA daily. More recently, cases of acute liver injury have been emerged in association with a product claimed to contain *Garcinia cambogia*. It is alarming that not only mild side effects (transient and moderate enzyme elevations) were reported but symptomatic acute hepatitis and acute liver failure were also described (LiverTox, 2012a). The frequency of hepatic adverse reactions is not known but it seems to be uncommon (<1:10,000). HCA might influence glucose homeostasis by modifying insulin sensitivity, and increasing gluconeogenesis and the formation of ketone bodies (McCarty and Majeed, 1994; Jena et al., 2002).

### *Hoodia gordonii*

The consumption of *Hoodia gordonii* (Masson) Sweet ex Decne. has its tradition among a native South African people (van Heerden, 2008). Bushmen used to eat this succulent plant for its appetite reducing effects. In Europe, *H. gordonii* can only be marketed after appropriate safety assessment, since it was not used as a food or food ingredient before 15 May 1997 (European Commission, 2020). An oxypregnane glycoside, P57 (16) is assumed to be responsible for the appetite reducing effects of *H. gordonii*. It was found that after intracerebroventricular administration, P57 increased ATP production in the hypothalamus (MacLean and Luo, 2004). No published, peer-reviewed meta-analysis of RCTs examining the efficacy of *Hoodia* were found in the literature (PubMed/Medline, the Cochrane Library, ClinicalKey and Google Scholar). In a placebo-controlled study involving overweight women, the weight loss efficacy of *H. gordonii* was compared to placebo. Participants were classified by body fat percentages, and 25 of them took *H. gordonii* and 24 received placebo (Blom et al., 2011). To ensure identical circumstances for each participant during the 15-day-long study, they stayed in the clinic 4 days prior to the study for a run-in period and during the 15-days treatment period. Participants were asked to consume a yogurt drink 1 h prior to each breakfast and dinner. The yogurt contained 1,110 mg *H. gordonii* or placebo. There were no serious adverse events but nausea, vomiting, and disturbances of skin sensation occurred in the verum group. Blood pressure, pulse rate, bilirubin and alkaline phosphatase levels increased significantly in the verum group. Recently alarming side effects (elevated blood pressure and heart rate) that are in line with the previously describe study have been reported (Roza et al., 2013).

### *Stevia rebaudiana*

The plant *Stevia rebaudiana* (Bertoni) Bertoni is native to South America, and Native Americans used it for centuries to sweeten their food and also for medicinal purposes, as herbal tea to alleviate several ailments, such as heartburn (Lemus-Mondaca et al., 2012). Glucosides obtained from *S. rebaudiana* are approximately 300 times sweeter than sucrose. Nowadays, when obesity has become a worldwide problem, low- and no-calorie sweeteners, such as *S. rebaudiana* offers an alternative that might help reduce sugar intake, and decrease the incidence of diseases derived from high refined sugar consumption (Samuel et al., 2018).

The whole plant and also the dried leaves of *S. rebaudiana* are novel foods in the EU based on the Regulation (EC) No 258/97 (European Commission, 1997). Extracts prepared from the leaves of *S. rebaudiana* are authorized as novel food, based on the Regulation (EC) No 1333/2008 on food additives or Regulation (EC) No 1334/2008 on flavorings, respectively (European Commission, 2008). The regulation covers only herbal tea containing or prepared with leaves of *S. rebaudiana* and preparations that are to be used for sweetening or flavoring purposes, every other use of *Stevia* is still unauthorized in the European Union.

*S. rebaudiana* appears to be safe. Human and animal studies have shown that steviol glycosides do not possess nor carcinogenic, nor mutagenic, nor teratogenic activates, and they do not have acute or subacute toxicity (Momtazi-Borojeni et al., 2017).

### *Acacia rigidula*

Extracts of *Acaciopsis rigidula* (Benth.) Britton and Rose leaves are used in weight loss products with little or no published clinical data about their potential biological effects, and has no documented history of use as food or traditional herbal treatment (Pawar et al., 2014). A comprehensive literature search in several databases (PubMed/Medline, the Cochrane Library, ClinicalKey and Google Scholar) yielded no results regarding its safety and efficacy. The consumption of *A. rigidula* might be dangerous because it contains appreciable amounts of toxic azotoids (Clement et al., 1998). *A. rigidula* is still not a novel food in the European Union, hence it cannot be market as food supplement, only taxons *Acacia arabica* (Lam.) Willd. *,* *Acacia nilotica*(L.) Delile*,* *Acacia senegal* (L.) Willd. *and Acacia verek* Guill. and Perr. are authorized as a novel food ingredient (European Commission, 2020)*.*

## Materials and Methods

Retrospective data were extracted from the RASFF portal. Data from individual warnings were recorded (date, product, product category, notification type, countries concerned, subject, action taken, distribution status and risk decision). Records were grouped into four main categories:

1. “A” for unauthorized ingredients;
2. “B” for unsafe ingredients;
3. “C” if there was a problem with the level of the ingredient (too high or too low);
4. “D” other problems (eg mislabeling, taste disturbance).

RASFF signals are classified as alert, information notification or border rejection as part of its RASFF Portal. Subcategories were created based on the intended use of the reported product. The risks and adverse effects were also assessed. Data from 1988 to 2019 were extracted from the reported supplements database on January 1, 2020. Each entry was individually reviewed. After the data set was categorized, descriptive analyses were performed using Microsoft Excel 2010 (Microsoft Excel, RRID:SCR_016137) for Windows (Microsoft Inc.).

## Results

The raw data set from the RASFF database included 2,559 records of food supplements with quality problems and several of these products were marketed to facilitate weight loss [319 (12.5%)]. 202 (63.3%) of these slimming products contained unapproved, synthetic weight loss pharmacons. Other frequently used adulterants were erectile dysfunction drugs and performance-enhancers which are not included in this article.

**FIGURE 1:**
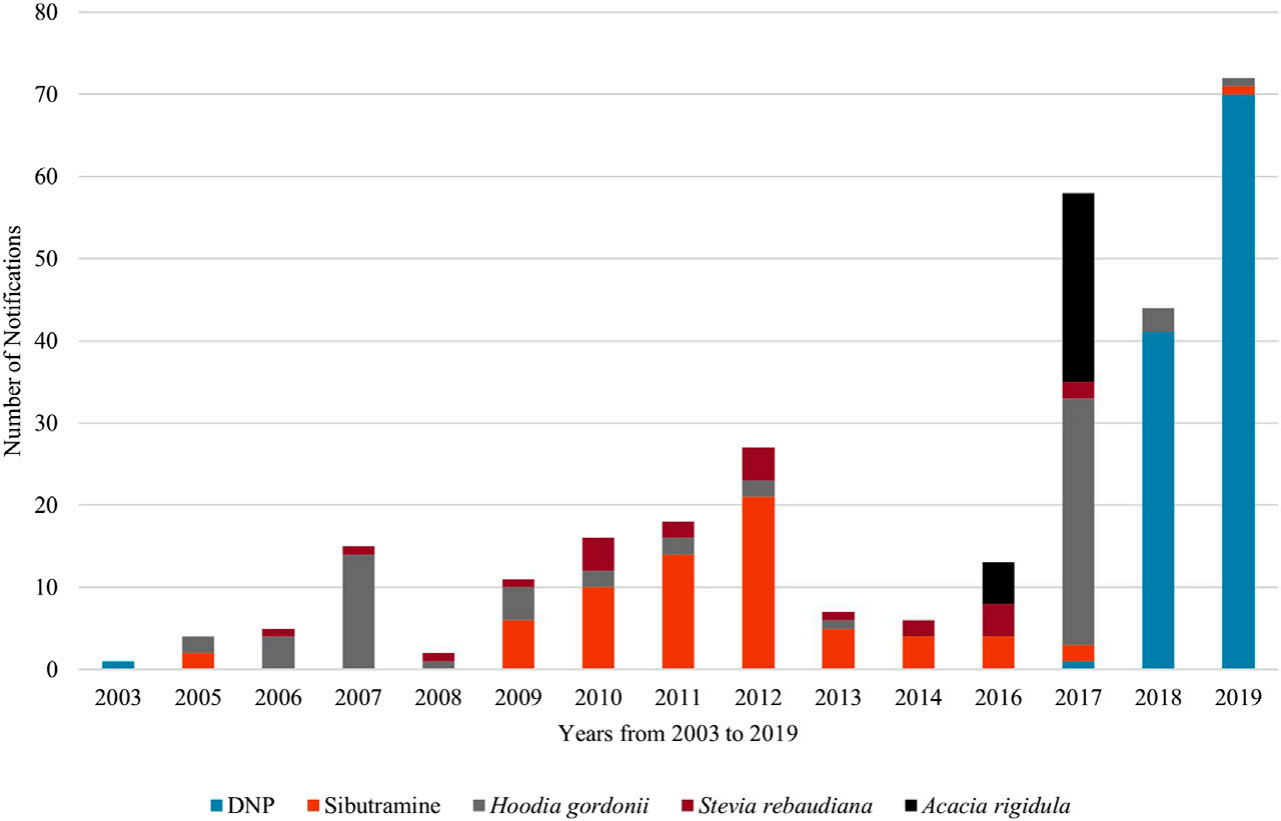
Notifications on weight loss food supplements in the RASFF (2003–2019).

The overall reports extracted from RASFF show that the first notifications were created in 2003, and the number of the reported signals kept growing until 2019 (especially in case of DNP). The majority of the adulterated anti-obesity products contained DNP (113 of 319, 35.4%). Sibutramine was the second most frequent adulterant (69 products, 21.6%) in the weight loss food supplement category and it was reported in almost every year, in contrast with DNP, which was reported only in four different years, in 2003, 2017, 2018, 2019. Phenolphthalein, a laxative with genotoxic and carcinogenic potential was the less common synthetic adulterant, with 20 reports. Unauthorized plant ingredients such as *Hoodia gordonii* (Masson) Sweet ex Decne., *Stevia rebaudiana* (Bertoni) Bertoni, and *Acaciopsis rigidula* (Benth.) Britton and Rose (in RASFF portal recorded as syn.: *Acacia rigidula*) were reported less frequently (Figure 1).

Based on our statistical overview, adulterated DNP products have been reported mainly in the United Kingdom. Whereas sibutramine has been reported with the highest number in Germany followed by Cyprus and Slovenia, it was reported less frequently in other countries (Supplementary Figure S1).

Based on the reports, DNP as adulterant first appeared in 2003, in Finland, and there were no reports on DNP until 2017, and then in 2017, it appeared again, and the number of DNP-containing products started to increase dramatically. The notifications originated from the United Kingdom and Cyprus.

Sibutramine was first detected in food supplements in the 2005, and since then it is a common adulterant in the EU. The number of reports on sibutramine-containing products peaked in 2012 (21 reports) and concerned many countries in the EU. The first 2 phenolphthalein reports originated from Hungary and Cyprus. The maximum number of phenolphthalein was seven reports in 2013 from Germany (Supplementary Table S4).

The ratio of the emergence of these three, commonly used synthetic compounds can be seen in Supplementary Figure S2. Based on the reports, it can be concluded that sibutramine emerged more frequently, but it occurred in fewer products. DNP was mainly present in 2018 (41 times) and in 2019 (70 times), while the popularity of sibutramine seemingly peaked in the early 2010s, in 2011 and 2012 14 and 21 reports were registered, respectively. Phenolphthalein emerged with the highest reports in 2012 with six reports and with seven reports in 2013.

Based on the RASFF signals *Hoodia gordonii* [66 of 117 (56.4%)], Stevia rebaudiana [23 (19.66%)] and *Acacia rigidula* [28 (23.93%)] were reported as unauthorized herbal products. The countries affected by adulterated products were Poland, Lithuania, France, Malta, Spain, Belgium, Austria, Switzerland, Ireland, Sweden, and Finland as shown in Supplementary Figure S3.

*Hoodia gordonii* was reported for the first time, in 2005 in the Netherlands (2 reports). The greatest number of products containing *Hoodia goordonii* was reported in 2017 (30 reports). In 2016, there were no reports on *Hoodia gordonii*. The first appearance of a product containing unauthorized *Stevia* *rebaudiana* was reported in Denmark in 2006. The highest number of reports on products containing unathorized *Stevia rebaudiana* was four in 2010. Except for 2005, 2015, 2018, 2019, it was present in every year from 1988–2019. *Acacia rigidula* was first reported in 2016, firstly in the Netherlands, but later also in Belgium, Austria, France, Malta, Spain and other European countries, overall 28 records were found in RASFF (Supplementary Table S5).

The ratio of these three reported, natural products with safety concerns are represented in Supplementary Figure S4. *Hoodia gordonii* was present more often, in smaller quantities, with the highest occurrence of 30 reports in 2017. *Stevia rebaudiana* has been reported in almost every year, but the highest number of reports was only four. The appearance of *Acacia rigidula* only started at 2016 and it emerged again in 2017 with 23 records.

## Discussion

The aim of our work was to summarize the trends concerning adulterated food supplements associated with a warning released by the RASFF between 1988 and 2019, focusing on products with intended use as slimming agent. RASFF is a platform for reporting food safety issues within the European Union. When a RASFF member suspects that a food or feed poses a serious risk to the people’s health, the member state should notify the European Commission (EC) via RASFF without any delay. In cases of withdrawing or recalling products from their market or in cases when rapid measures are needed, the members are obliged to notify the EC to help protect peoples’ health.

The increasing number of signals on illegal food supplements in RASFF reveals that the presence of undeclared ingredients poses an important public health concern. Illegal supplements marketed for weight loss were most commonly adulterated with DNP or sibutramine between 1988 and 2019. The use of former one may result in quick weight loss, but often causes serious adverse events (Colman, 2007). Several deaths attributable to DNP have been published (Cann and Verhulst, 1960). DNP was detected as an adulterant for the first time in 2003, but the number of products containing this compound has been increasing from 2017 through 2019, reported mainly in the United Kingdom. Sibutramine was reported in several countries; however, the number of products containing sibutramine was lower. SCOUT confirmed that sibutramine (at daily doses ranging from 10 to 15 mg) increases the risk for nonfatal myocardial infarction and nonfatal stroke in patients with preexisting cardiovascular disease, and have an increased potential to develop high blood pressure or pulse rate (Sharma et al., 2009).

It is alarming that the majority of the reported signals were in connection with unsafe synthetic substances. There have been increasing number of reports on DNP, and since this substance can cause serious side effects it is necessary to monitor the use of DNP more closely in the future.

Out of the most popular food supplements with natural origin the extracted materials of *Hoodia gordonii* (Masson) Sweet ex Decne., *Stevia rebaudiana* (Bertoni) Bertoni, and *Acaciopsis rigidula* (Benth.) Britton and Rose were unauthorized products registered in RASFF from 1988–2019. *Stevia rebaudiana* seems to be the least dangerous component based on the reports of our review on RASFF. It was reported in small quantity, and for now became authorized as a novel food according to EC Regulation EC No. 258/97 (European Commission, 1997). In spite of that it has been traditionally used for hundreds of years, more scientific and clinical studies are needed to verify its safety, because it was represented almost in every year in RASFF from 1988–2019, and it is very popular among the consumers.

The other two plants (*H. gordonii*, *A. rigidula*) are still not authorized as a novel food and their safety is not scientifically proven (Roza et al., 2013; Clement et al., 1998).

Despite the fact that *H. gordonii* is often used as an adulterant, and advertised for its weight loss promoting effects, there is still little known about its chemical constituents and their mechanism of action. Recent research suggests that the use *H. gordonii* may cause increased blood pressure and pulse rate (Roza et al., 2013). Taken into consideration that *H. gordonii* emerges regularly from 1988, it would be important to monitor food supplements containing *Hoodia.*

*A. rigidula* is a shrub native to the Southeastern United States, and it contains several biogenic amines. The plant has been marked in products promoting weigh loss; however, its effects are not yet supported by either its traditional usage, since it has never been used in the traditional medicine, or by research results. *Acacia rigidula* occurred in the RASFF portal recently, the presence of *A. rigidula* should be monitored closely in weight loss dietary supplements.

## Conclusion

As several medications used to manage body weight are no longer available on the market, because of their serious adverse effects; there is a clear need for effective products to support weight loss because currently there are only a few therapeutic options to address this issue. However, the efficacy of natural ingredients usually does not meet the customers’ expectations.

Some products (typically sold as food supplements) are adulterated with synthetic compounds to increase their efficacy. Adulterated food supplements may cause serious adverse effects, and their interactions with other medicines are also unpredictable. Therefore, it is alarming that the number of signals on adulterated products in RASFF is increasing. As the food supplement industry continues to grow worldwide, it is important to mark these signals as a public health issue, and to elaborate various measures to decrease the number of these signals by improving the safety, quality and testing of food supplements.

## Author Contributions

DK. collected and analyzed the data and drafted the manuscript. BT and OR. analyzed the data and checked the manuscript for validity. DC. conceptualized the research and did the final check of the manucsript.

## Funding

The financial support of University of Szeged Open Access Fund is acknowledged.

## Conflict of Interest

The authors declare that the research was conducted in the absence of any commercial or financial relationships that could be construed as a potential conflict of interest.

## Abbreviations

5-HT2C, 5-hydroxytryptamine; ATP, adenosine triphosphate; BMI, body-mass index; CB1-receptor, cannabinoid receptor type 1; CHMP, Committee for Medicinal Products for Human Use; CLA, conjugated linoleic acid; DNP, 2,4-dinitrophenol; EC, European Commission; EGCG, epigallocatechin-3-gallate; EMA, European Medicines Agency; EU, European Union; FA, fatty acid; GCE, green coffee extract; GLP-1, glucagon like peptide-1; GM, glucomannan; HCA, (-)-hydroxycitric acid; NA, noradrenaline; NR, no reports; POMC, pro-opiomelanocortin; PPA, phenylpropanolamine; RASFF, rapid alert system for food and feed; RCT, randomized controlled trial; SCOUT, sibutramine cardiovascular outcomes trial; T2DM, type 2 diabetes mellitus; WMD, weighted mean difference.
