# Supplementary material for: A Systematic Review of the European Rapid Alert System for Food and Feed: Tendencies in Illegal Food Supplements for Weight Loss
Source: Front Pharmacol. 2021 Jan 26;11:611361. doi: 10.3389/fphar.2020.611361 (PMC7870490; doi:10.3389/fphar.2020.611361)
Supplement: Supplementary file 1 [file datasheet1.docx]

**Supplementary Material**

**Table S1** Available pharmacological treatment possibilities for obese patients in the European Union.

| **Compound** | **Approved in the EU** | **Mechanism of action** | **Possible adverse effects** |
| --- | --- | --- | --- |
| **Orlistat** | since 1998 | Inhibit intestinal lipases | liver damage, vitamin deficiency |
| **Liraglutide** | since 2009 | appetite suppressant, and delays gastric emptying | hypoglycemia, diarrhea, cardiovascular outcomes |
| **Naltrexone-bupropion** | since 2015 | appetite suppressant /antidepressant | headache, constipation, possible cardiovascular side effects |
| **Phentermine** | 1956–2012 | appetite suppressant | cardiovascular and central nervous system effects |

* based on the authorization of the national competent authority, phentermine resinate is available in modified-release capsules in the Czech Republic

**Table S2** Withdrawn amphetamine type medications of obese patients in the European Union.

| **Ingredient** | **Approved in the EU** | **Mechanism of action** | **Possible adverse effects** |
| --- | --- | --- | --- |
| **Amphetamine** | 1932–1968 | increases noradrenaline, serotonine, and dopamine lrelease | dependence, cardiac complications |
| **PPA** | 1939–1997 | increases noradrenaline, serotonine, and dopamine release | haemorrhagic stroke |
| **Fenfluramine, dexfenfluramine** | 1963–1997,  1985–1997 | increase serotonine release | pulmonary hypertension |
| **Fenfluramine, phentermine (fen-phen)** | 1984-1997 | increase serotonine release, appetite suppressant | pulmonary hypertension |

**Table S3** Withdrawn non-amphetamine derivative type medications of obese patients in the European Union.

| **Ingredient** | **Approved in the EU** | **Mechanism of action** | **Possible adverse effects** |
| --- | --- | --- | --- |
| **DNP** | 1933–1938 | increase thermogenesis | hyperthermia |
| **Rimonabant** | 2006–2009 | CB_1_-receptor blocker | depressed mood, anxiety, and suicidal ideation |
| **Sibutramine** | 1999–2010 | antidepressant, appetite suppressant | increased systolic and diastolic blood pressure and heart rate |
| **Lorcaserin** | 2012–2013 | increases serotonin release | increased occurrence of cancer |

**Table S4** Notifications on DNP and sibutramine.

| YEAR | DNP | | sibutramine | |
| --- | --- | --- | --- | --- |
| 2003 | 1 | Finland | 0 | NR* |
| 2004 | 0 | NR* | 0 | NR* |
| 2005 | 0 | NR* | 2 | Germany, Poland |
| 2006 | 0 | NR* | 0 | NR* |
| 2007 | 0 | NR* | 0 | NR* |
| 2008 | 0 | NR* | 0 | NR* |
| 2009 | 0 | NR* | 6 | Romania, Germany, Italy, Cyprus |
| 2010 | 0 | NR* | 10 | Cyprus, Germany, Italy, Romania, Slovenia |
| 2011 | 0 | NR* | 14 | Cyprus, Romania, Hungary, Germany, Malta |
| 2012 | 0 | NR* | 21 | Belgium, Bulgaria, Germany, Lithuania, Luxembourg, Slovenia |
| 2013 | 0 | NR* | 5 | Belgium, Cyprus, Germany |
| 2014 | 0 | NR* | 4 | France, Germany |
| 2015 | 0 | NR* | 0 | NR* |
| 2016 | 0 | NR* | 4 | Germany, Spain, Cyprus |
| 2017 | 1 | United Kingdom | 2 | Cyprus, Slovenia |
| 2018 | 41 | United Kingdom | 0 | NR* |
| 2019 | 70 | United Kingdom, Cyprus | 1 | Germany |

* no reports

**Table S5** Unauthorized *Hoodia gordonii* and *Stevia* rebaudiana and *Acacia rigidula* products.

| YEAR | *Hoodia gordonii* | | *Stevia rebaudiana* | | *Acacia rigidula* | |
| --- | --- | --- | --- | --- | --- | --- |
| 2005 | 2 | Netherlands | 0 | NA* | 0 | NR* |
| 2006 | 4 | Denmark, Finland, Malta | 1 | Denmark | 0 | NR* |
| 2007 | 14 | Malta | 1 | Malta | 0 | NR* |
| 2008 | 1 | Germany | 1 | Slovenia | 0 | NR* |
| 2009 | 4 | Slovenia, Malta | 1 | Slovakia | 0 | NR* |
| 2010 | 2 | Ireland, Lithuania | 4 | Hungary, Belgium, Cyprus | 0 | NR* |
| 2011 | 2 | Belgium, Germany | 2 | Malta, Finland | 0 | NR* |
| 2012 | 2 | France, Finland | 4 | Lithuania, France, Finland | 0 | NR* |
| 2013 | 1 | Malta | 1 | Lithuania | 0 | NR* |
| 2014 | 0 | NR* | 2 | Malta, Finland | 0 | NR* |
| 2015 | 0 | NR* | 0 | NR* | 0 | NR* |
| 2016 | 0 | NR* | 4 | Poland, Spain, Malta | 5 | Netherlands, Poland, United Kingdom, Germany |
| 2017 | 30 | Poland, France, Malta, Netherlands, Belgium, Denmark, Switzerland, Cyprus, Ireland, Sweden | 2 | Slovakia, Spain | 23 | Poland, Lithuania, France, Malta, Spain, Belgium, Austria, Switzerland, Ireland, Sweden |
| 2018 | 3 | Spain, Sweden | 0 | NR* | NR* | NR* |
| 2019 | 1 | Sweden | 0 | NR* | NR* | NR* |

* no reports

Figure S1. Notifications on food supplements containing DNP or sibutramine from 2003 to 2019.

**Figure S2.** Ratio of DNP (inside) and sibutramine (outside) from 2003 to 2019.

Figure S3. Unauthorized *Hoodia gordonii*, *Stevia rebaudiana* and *Acacia rigidula* products from 2003 to 2019.

**Figure S4**. Ratio of *Hoodia gordonii*, (outside circle) and *Stevia rebaudiana* (middle circule) and *Acacia rigidula* (inside circle) from 2003 to 2019.
