# Supplementary figures and images for: A Systematic Review of the European Rapid Alert System for Food and Feed: Tendencies in Illegal Food Supplements for Weight Loss
Source: Front Pharmacol. 2021 Jan 26;11:611361. doi: 10.3389/fphar.2020.611361 (PMC7870490; doi:10.3389/fphar.2020.611361)

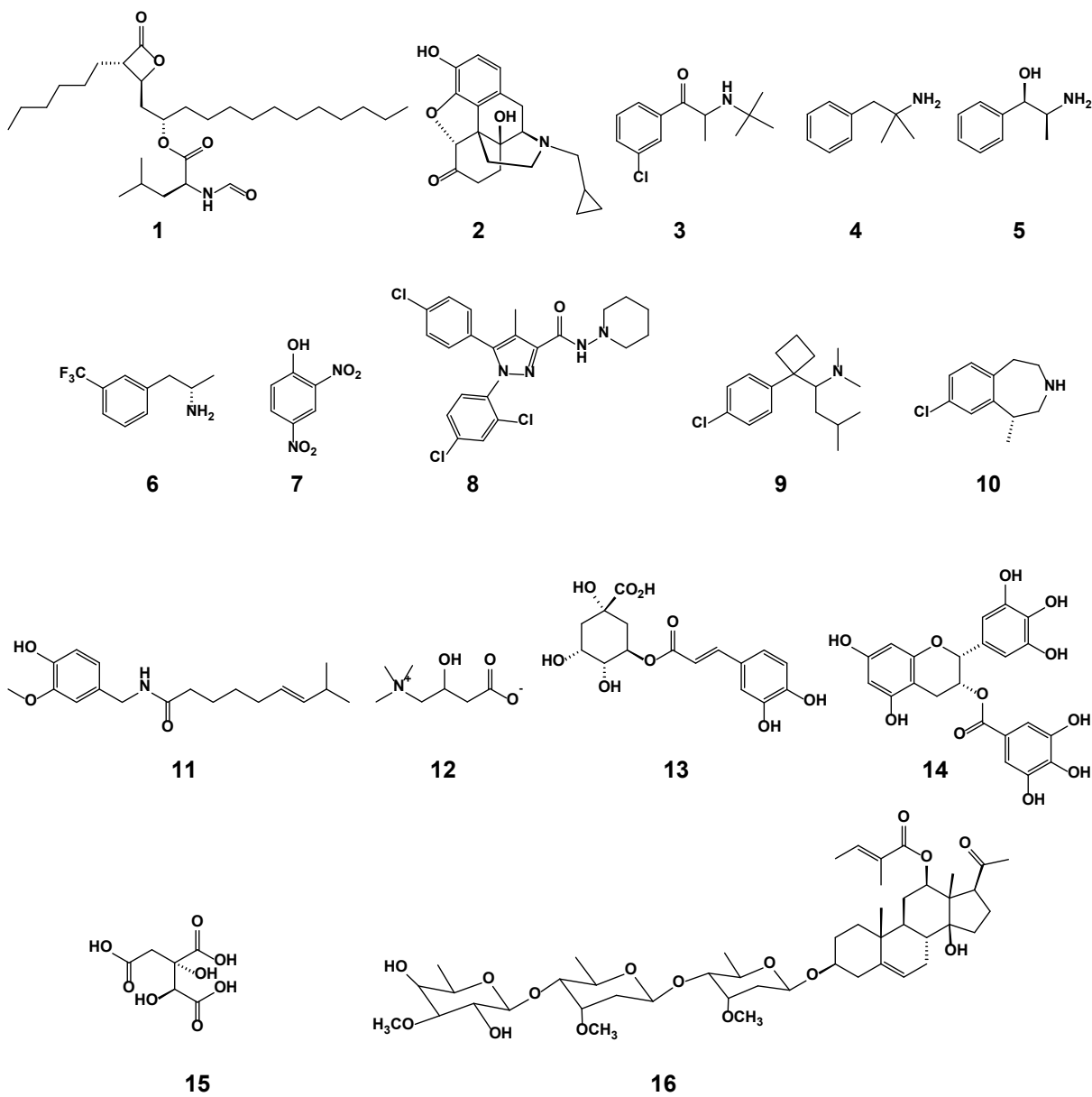

Supplement: Supplementary file 2 [file image1.pdf]
